# Oviposition and Sex Ratio of the Redbanded Stink Bug, *Piezodorous guildinii*, in Soybean

**DOI:** 10.3390/insects7020027

**Published:** 2016-06-17

**Authors:** Joshua H. Temple, Jeffrey A. Davis, Jarrod T. Hardke, Paul P. Price, B. Rogers Leonard

**Affiliations:** 1 DuPont Crop Protection, Bradenton Field Station, 3301 51st St. East, Bradenton, FL 34208, USA; joshua.h.temple@dupont.com; 2 Department of Entomology, Louisiana State University Agricultural Center, 404 Life Sciences Building, Baton Rouge, LA 70803, USA; 3 Department of Crop, Soil, and Environmental Sciences, University of Arkansas, 2900 Hwy. 130 E., Stuttgart, AR 72160, USA; jhardke@uaex.edu; 4 Macon Ridge Research Station, Louisiana State University Agricultural Center, 212A Macon Ridge Rd., Winnsboro, LA 71295, USA; pprice@agcenter.lsu.edu; 5 LAES Administration, Louisiana State University Agricultural Center, 104 J. N. Efferson Hall, Baton Rouge, LA 70803, USA; rleonard@agcenter.lsu.edu

**Keywords:** Hemiptera, Pentatomidae, soybean phenology, maturity group, life history

## Abstract

Redbanded stink bug, *Piezodorus guildinii* (Westwood), is a significant soybean pest across the mid-south region of the United States. The objectives of these studies were to characterize: (1) redbanded stink bug oviposition in relationship to soybean maturity group (MG), plant structure, crop phenology, and vertical distribution within the plant canopy; and (2) redbanded stink bug adult sex ratios in relationship to soybean phenology. A total of 5645 redbanded stink bug eggs in 421 egg masses (clusters) were field collected from naturally-occurring populations in MG IV and V soybean over a three year period (2009 to 2011). The mean number of eggs within a cluster was 16.6 ± 0.3. Plant structures by MG interactions were highly significant with more egg masses oviposited on leaves in MG IV (79.4%) and more on pods in MG V (72.7%). The ratio of females to males was similar in all soybean growth stages except R5, where the sex ratio increased to 1.4:1, coinciding with peak oviposition. Only 29.9% of egg clusters in MG IV and 18.3% of egg clusters in MG V were oviposited in the upper 35 cm of the soybean canopy. Based on these results, sampling strategies and insecticide application placement for stink bugs may require modification.

## 1. Introduction

Several phytophagous stink bugs including green stink bug, *Chinavia hilaris* (Say), southern green stink bug, *Nezara viridula* L., and the brown stink bug complex, *Euschistus* spp., are annual pests in many Southern USA soybean fields. In 2002, an emerging stink bug pest, the redbanded stink bug, *Piezodorus guildinii* (Westwood) exceeded action threshold levels in Louisiana and has since become the most yield-limiting stink bug pest in the Louisiana soybean production system [1]. A comprehensive survey of stink bugs in Louisiana soybean from 2008 to 2010 showed that the redbanded stink bug was the predominant (>50%) species [1]. The expansion of this pest’s range has not been limited to Louisiana. Redbanded stink bugs have now reached pest status for soybean in all states bordering Louisiana and have been reported as far north as Missouri and Tennessee [1].

The redbanded stink bug is a neotropical stink bug species that ranges from Argentina north to the Southern United States [2]. The redbanded stink bug is a significant annual pest in South American soybean, especially in Brazil [3]. In the United States, this species was first reported during the 1960s and has been documented in several states including South Carolina, Florida, Georgia, and New Mexico [3]. Though having been previously reported on soybean in the United States, it was not considered an economic pest [4].

Studies on nymphal development and survivorship, adult longevity and reproduction, and life stage dispersal on South American soybean have provided considerable information on the biology and ecology of the redbanded stink bug [5]. Even though redbanded stink bug is highly injurious to soybean, it reproduces poorly and has shorter longevity on soybean plants compared to other non-crop hosts [5]. The number of egg clusters per female ranges from three clusters on soybean to as many as 37 on *Indigo* spp. [5]. Panizzi and Smith [6] characterized the oviposition, development time, sex ratio, and longevity of redbanded stink bug on soybean in Brazil. This study reported that the average number of eggs were 15.1 per cluster and 60% of the clusters were observed on pods. The sex ratio observed in these field studies was 1.4 females for each male [6]. Redbanded stink bug eggs maintained in the laboratory at 24 °C and 80% RH required a mean of 7.5 days from oviposition to eclosion. In a subsequent study, Link and Concatto [7] found number of eggs per clusters (mean = 17.5) ranged from 4 to 39. Oviposition preference among soybean pods, stems, and leaves indicated the greatest frequency of eggs occurred on pods (80%) in that study. Silva and Ruedell [8] reported that redbanded stink bug oviposited 51% of eggs on pods and 48% on leaves. No studies have examined the vertical distribution of redbanded stink bug eggs within the soybean plant canopy or oviposition frequency related to soybean phenology.

Limited information is available describing the biology of redbanded stink bug in U.S. soybean agro-ecosystems. Compared to other stink bugs attacking soybean, this pest is less susceptible to insecticides commonly used on soybean for stink bug control [9,10]. This aspect coupled with limited knowledge of pest biology and wild host range has hindered satisfactory management. The redbanded stink bug has also shown a propensity to develop high infestations (five-fold action threshold) within soybean fields over a relatively brief period [1]. Knowledge of oviposition characteristics and adult behavior during soybean phenological growth stages is needed to develop effective IPM strategies for controlling this pest. The objectives of these studies were to characterize: (1) redbanded stink bug oviposition in relationship to soybean maturity group (MG), plant structure, crop phenology, and vertical distribution within the plant canopy; and (2) redbanded stink bug adult sex ratios in relationship to soybean phenology.

## 2. Materials and Methods

### 2.1. Oviposition Surveys

Observations were made on non-insecticide treated soybean at four Louisiana State University Agricultural Center Research Stations replicated over three years; Ben Hur Research Station (East Baton Rouge Parish, Baton Rouge, LA, USA, 2009 to 2011), New Iberia Research Station (Iberia Parish, Jeanerette, LA, USA; 2009 to 2011), Dean Lee Research Station (Rapides Parish, Alexandria, LA, USA; 2010 to 2011) and Macon Ridge Research Station (Franklin Parish, Winnsboro, LA, USA; 2009 to 2011). These stations are spread throughout the soybean growing region of Louisiana and cover a wide geographic area within the state. Two maturity groups (MG) soybeans, AsGrow 4606 (MG IV) and AsGrow 5606 (MG V), were planted in large contiguous blocks (0.1 ha each) at planting dates recommended by the Louisiana Cooperative Extension Service [11]. Naturally-occurring stink bug populations were allowed to infest field plots.

Sampling for redbanded stink bug eggs was initiated at the R2 (full flower) stage and continued weekly until R7 (beginning maturity). On each sampling date, soybean growth stages for each MG were recorded based on descriptions by Fehr *et al.* [12]. Thirty plants per location per week were randomly removed from each MG. Whole plants were destructively sampled and examined for the presence of redbanded stink bug egg clusters. Specifically, data on growth stage, plant height (cm), number of nodes per plant, oviposition site (leaf [abaxial or adaxial], pod, or stem), main stem node of oviposition site, and number of eggs per cluster (mass) were recorded. Based on position within the vertical plant strata, egg clusters were further categorized as being in the upper, middle, or lower plant canopy.

### 2.2. Adult Sex Ratio

Observations were made on non-insecticide treated soybean at two Louisiana State University Agricultural Center Research Stations replicated over two years; Ben Hur Research Station (East Baton Rouge Parish, Baton Rouge, LA, USA; 2008 to 2009) and Macon Ridge Research Station (Franklin Parish, Winnsboro, LA, USA; 2008 to 2009). Commercial soybean varieties AsGrow 4404 (MG IV; 2008), AsGrow 5905 (MG V; 2008), AsGrow 4606 (MG IV; 2009) and AsGrow 5606 (MG V; 2009), were planted in large contiguous blocks (0.1 ha each) at planting dates recommended by the Louisiana Cooperative Extension Service [11]. Each MG was divided into six sub-plots. Each of the six sub-plots within a MG was sampled weekly from R1 (beginning bloom) to R8 (physiological maturity) using a standard (38 cm diameter) sweep net and taking 25 sweep samples (150 sweeps total/MG/week). Individual rows that were sampled within each sub-plot were alternated each week so that a row was not sampled more than once within a period of four weeks. On each sampling date, the soybean growth stages for each MG were recorded. Each individual set of sweep net samples was bagged, labeled, transported to the laboratory, and frozen until it could be evaluated. Samples of redbanded stink bug adults were segregated and sex was determined based on diagnostic keys [3].

### 2.3. Data Analysis

Data were tested for normality using the Kolmogorov-Smirnov test in PROC CAPABILITY and tested for homogeneity using the Levene Test for Homogeneity of Variances in PROC GLM [13]. As the number of egg clusters varied by year, data were converted into proportions which were arcsine square-root transformed. Data were then analyzed by two-way analysis of variance (ANOVA) using PROC MIXED [13] with MG and oviposition site as two main factors replicated within a year by location. Means were separated using Tukey’s Honestly Significant Difference (HSD) test at a = 0.05 level. One-way ANOVA using PROC MIXED determined differences within each MG for plant height, number of nodes per plant, oviposition site, growth stage, and vertical strata. Means were separated using Tukey’s HSD test at a = 0.05 level. Chi-square analysis was used to compare the frequency of males and females collected within a given soybean phenological stage (PROC FREQ, [13]).

## 3. Results

### 3.1. Oviposition Surveys

A total of 5,645 redbanded stink bug eggs in 421 egg clusters were sampled and characterized over three years. The number of egg clusters and eggs per cluster did not differ by location (*p* = 0.822) or by MG (*p* = 0.535). The mean number of eggs per cluster was 16.6 ± 0.3. The number of eggs within a cluster ranged from 2 to 55. Plant heights were different between MG; 77.6 ± 0.5 cm for MG IV and 92.9 ± 0.4 cm MG V (*F*_1,2_ = 201.70; *p* ≤ 0.001). Within a MG, plant heights differed by growth stage (Table 1). Tallest plant heights within MG IV occurred at R5 to R8 with shortest at R2 (Table 1). Within MG V soybean, tallest plant heights occurred at R7 and shortest at R2 (Table 1). Number of nodes per plant was different between MG; 13.3 ± 0.1 for MG IV and 12.4 ± 0.1 for MG V (*F*_1,2_ = 51.90; *p* ≤ 0.001). The most number of nodes within MG IV occurred at R5 to R8 with least at R2 (Table 1). For MG V, the greatest number of nodes occurred at R7 and least at R2 (Table 1).

**Figure 1:**
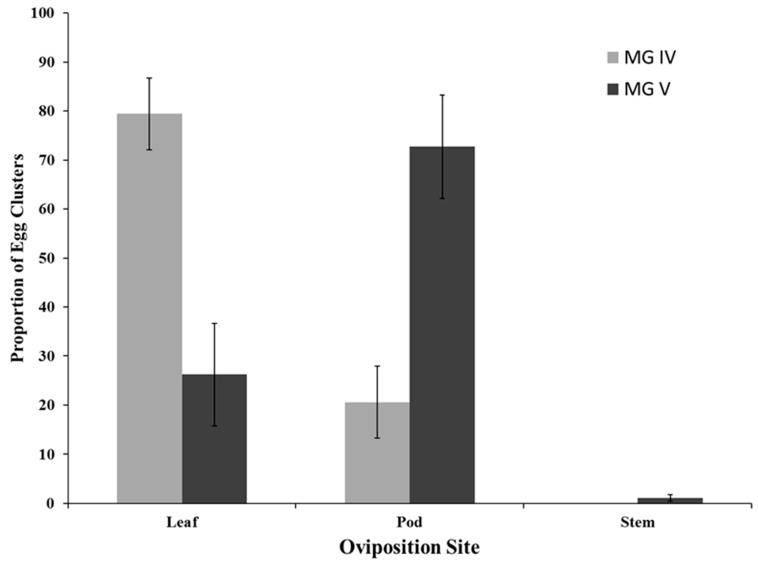
Frequency of redbanded stink bug egg clusters by oviposition site within MG IV and MG V soybeans.

Interactions between MG and oviposition site were highly significant (*F*_2,2_ = 18.90; *p* ≤ 0.001). In MG IV, the majority of the egg clusters were deposited on the leaves (*F*_2,2_ = 33.91; *p* ≤ 0.001) (Figure 1) and in the MG V, most egg clusters were deposited on the pods (*F*_2,2_ = 6.04; *p* = 0.006) (Figure 1). Oviposition occurred on both the abaxial (top) and adaxial (bottom) leaf surfaces. In the MG IV soybeans, the proportion of egg clusters found on abaxial and adaxial surfaces was not statistically different; 67.5 ± 11.7% *vs.* 32.5 ± 11.7%, respectively (*F*_2,2_ = 1.35; *p* = 0.291). In the MG V soybeans, the proportion of egg clusters was equally distributed on both leaf surfaces; 56.4 ± 4.6% *vs.* 43.6 ± 4.6%, respectively (*F*_2,2_ = 1.18; *p* = 0.346).

**Figure 2:**
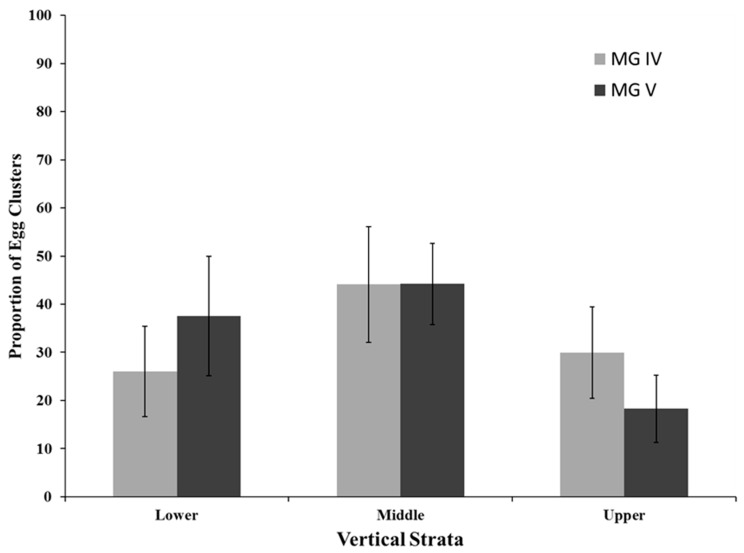
Oviposition site preference of redbanded stink bug within vertical strata in MG IV and MG V soybean.

Egg clusters were scattered throughout the upper, middle, and lower vertical strata (Figure 2) and were evenly distributed throughout the canopy in both MG IV (*F*_2,2_ = 0.87; *p* = 0.428) and MG V (*F*_2,2_ = 1.41; *p* = 0.278) soybeans. Egg clusters were collected during all soybean reproductive stages sampled, R2 (full flower) to R7 (beginning maturity); and were found at highest numbers in R5 to R7 stages (Table 2). In MG IV soybean, oviposition was at its lowest at the R3 stage and peaked during the R5 stage (Table 2). Similarly, in the MG V soybean, oviposition peaked during the R5 stage with the lowest oviposition occurring during R2 and R3 growth stages (Table 2).

### 3.2. Adult Sex Ratio

**Figure 3:**
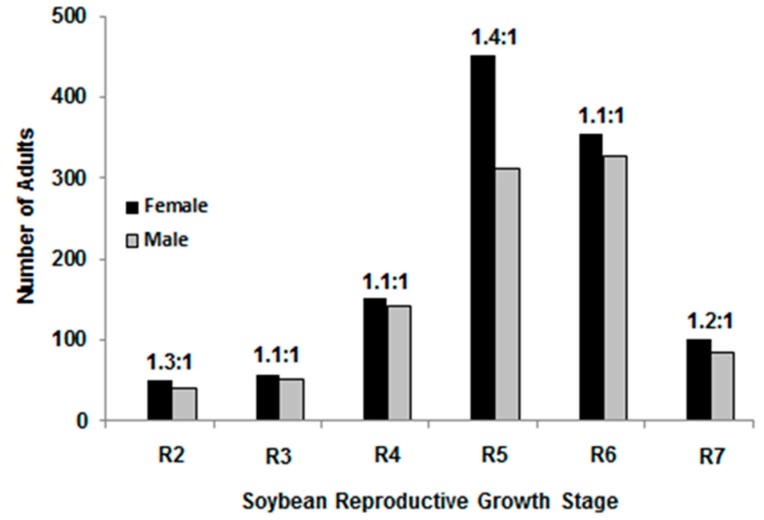
Redbanded stink bug sex ratio during soybean reproductive growth stages.

The sex ratio of 2,147 redbanded stink bugs collected at Winnsboro from 2008 to 2009 was 1.2 females to 1 male. Across all soybean reproductive growth stages, adult sex ratios ranged from 1.1 females to 1 male to 1.4 females to 1 male (Figure 3). The number of females compared to males was only significantly (χ^2^ = 25.32, *p* ≤ 0.001) higher during the R5 growth stage.

## 4. Discussion

The number of eggs per cluster averaged 16.6 across all samples in the current study. These results are similar to previous reports in Brazil with mean eggs per cluster ranging from 14.2 to 17.5 [6,7,8,14]. The range of eggs per cluster in the current study was from 2 to 55 and was similar to that in other studies (1 to 55 per cluster) [6,7,8]. The range of redbanded stink bug eggs per cluster is generally lower than that reported for the southern green stink bug (40 to 116 eggs per cluster), the predominant pest species in Southern U.S. soybean [15,16]. Eggs per female also vary between the two species on soybean, with redbanded stink bug having 28 to 80 eggs per female while southern green stink bug having 68 to 204 eggs per female [17].

In the current study, the primary oviposition sites for redbanded banded stink bug egg clusters were leaves and pods. This differed from past soybean surveys by Panizzi and Smith [6] and Link and Concatto [7], in which pods were the primary oviposition site, ranging from 60 to 80% of total egg masses. Silva and Ruedell [8] reported a more even distribution of egg clusters between leaves (51%) and pods (48%). In common bean, Link *et al.* [18] found over 90% of egg clusters were found on the adaxial side of leaves with only 3% on pods. In lentils, Link [19] found 20% of egg clusters on pods and 45% on leaves with the rest oviposited on tendrils, stems, and petioles. Clearly, plant type and architecture have a role in oviposition [19].

Oviposition on leaves is a behavior typically observed for naturally-occurring stink bugs (southern green, green, and brown stink bugs) of Louisiana soybean which prefer to oviposit on the abaxial surface [15,16,20,21]. In the present study, redbanded stink bugs oviposited exclusively on leaves until soybean plants reached the R4 stage (pod elongation). Leaves were still the predominant oviposition site during the later stages of development (R5 to R7 stages) in MG IV cultivars, but pods were the predominant oviposition site during the R5 to R7 stages of development in MG V cultivars. This difference could be related to differences in the growth habits of MG IV (indeterminate) and MG V (determinate) cultivars. However, native stink bugs prefer leaves and rarely oviposit on pods. Redbanded stink bug lays eggs in two rows while the other soybean pest stink bugs lay eggs in more than two rows [3]. Since redbanded stink bug oviposit on pods, nymphs can readily feed on young beans, potentially causing more seed damage more quickly.

Within the soybean plant canopy, 29.9% of egg clusters in MG IV and 18.3% of egg clusters in MG V were oviposited in the upper plant strata. These results suggest that redbanded stink bug adults prefer ovipositing in the lower two-thirds of the canopy. Previous redbanded stink bug oviposition surveys have not reported a within canopy preference. This behavior may be unique among the stink bugs in Louisiana soybean as southern green, green, and brown stink bugs prefer to oviposit in the upper canopy of soybean [15,16,20,21].

The most common protocol for sampling stink bugs in soybean relies on a sweep net (38 cm diam) that measures infestations in the upper canopy, a technique which is considered an appropriate sampling method for estimating phytophagous stink bug populations in soybean [22] primarily due to sampling efficiency [23]. Russin *et al.* [24] demonstrated that a complex of stink bugs (southern green, green, and brown) preferred to feed on pods in the upper half of the plant canopy. Because southern green, green, and brown stink bugs prefer ovipositing and feeding in the upper plant canopy, current sweep net sampling protocols work well to measure populations of both nymphs and adults. However, due to its oviposition behavior, sweep samples exclusively in the upper canopy may underestimate redbanded stink bug adults and nymphs, especially during the later soybean reproductive stages (R5 to R7). Future studies should evaluate other sampling methods such as shake sheets (beat cloths), which measure the entire plant canopy, to determine the optimal sampling strategy for this pest. If a sweep net is used, samples should be taken throughout the canopy and especially in the lower plant canopy of indeterminate soybean varieties which initially set pods in the lower portions of the canopy.

Historically, effective control of stink bugs in soybean has been through pesticide applications. With a high frequency of egg clusters found in the lower two-thirds of the plant canopy, redbanded stink bug nymphs could have less exposure to insecticide residues. The effectiveness of any chemical control measure is contingent on delivery of pesticide droplets at or near the target site. Hutchins and Pitre [25] reported that insecticide droplet deposition significantly decreased in the middle (37%) and lower plant canopy (82%) in conventional wide-row (96.5 cm spacing) soybean compared to that in the upper plant canopy. The introduction of glyphosate-tolerant soybean cultivars has resulted in an increase in conservation tillage soybean and adoption of narrow row (<76 cm row spacing) soybean systems. In narrow row (17.8 cm spacing) soybean, droplet depositions decreased more sharply in the middle (73%) and lower (84%) plant canopies when compared to conventional row soybean [25]. In arecent soybean study on 38 cm spacing, Barbosa *et al.* [26] found that regardless of spray rate or ground speed, spray deposition was “highly variable” with most deposited in the upper canopy. Redbanded stink bug nymphs in the lower plant canopy will less likely encounter insecticide residues. To compound this issue, many producers, in the interest of saving time and fuel costs, currently apply lower volumes of water with insecticide applications. This could all be contributing to the lack of effective control of this stink bug species and its increased insecticide tolerance [10]. To compensate, producers will need to increase insecticide canopy penetration and spray volume. Studies in Brazil suggest that chemical control of redbanded stink bug is greatly enhanced when insecticide application volumes are increased [27].

The adult sex ratio of redbanded stink bug observed in the current study was 1.2 females to 1 male. In Brazilian studies by Silva and Ruedell [8] and Panizzi and Smith [6], sex ratios for redbanded stink bug were 1.2:1 and 1.4:1, respectively. However, these were not season-long surveys. In the current study, the ratio of females to males was similar in all soybean growth stages except R5, where the sex ratio increased to 1.4:1, which coincided with peak oviposition. Insecticide applications applied at this time could impact population growth rates by reducing more females and thus limiting egg deposition.

This study is the first to report oviposition behavior of redbanded stink bug populations and the relationship to soybean phenology in the United States. The redbanded stink bug has become the predominant pest in Louisiana soybean within a decade after its first detection [1]. This pest appears to have a narrow non-crop host range, and soybean is one of the few available food sources after spring alternate hosts senesce. The redbanded stink bug has the propensity to develop large populations (3 to 5× action thresholds) very quickly in soybean [1]. Redbanded stink bug oviposit fewer eggs per cluster in soybean than the southern green, green, and brown stink bug, so oviposition frequency is not likely the sole factor creating these large populations [5]. Optimal nutritional sources are also not contributing to large redbanded stink bug populations. Redbanded stink bug survives very poorly on soybean, with only 12% to 42% reaching adulthood compared to the 70% to 98% of southern green stink bugs [17]. However, redbanded stink bug does reach the adult stage more quickly on soybean, 20 days to 23 days compared to 22 days to 28 days for southern green stink bug [17]. Redbanded stink bug may migrate into soybean fields at higher rates than other species. Another possibility for the large reproductive capacity of redbanded stink bugs in Louisiana soybean could be due to a lack of natural enemies. Future studies should evaluate predation and parasitism levels on redbanded stink bug adults, nymphs, and egg clusters.

The redbanded stink bug has become a serious pest of soybean in a relatively short time in Louisiana. With limited alternative hosts available to redbanded stink bug populations during the summer months, populations remain concentrated in soybean fields and are capable of quickly building to economically important levels. Historically, Louisiana has planted later-maturing varieties (MG’s V, VI, and VII) but began a transition to more of an early soybean production system during the late 1990’s to early 2000’s [28,29]. Interestingly, this transition to early soybean production systems has coincided with the increase in pest status of the redbanded stink bug. Additional research is needed to fully understand the biology of redbanded stink bug in Louisiana soybean agro-ecosystems and to determine how this species became the dominant pest of soybean within a decade.

## 5. Conclusions

In conclusion, plant structure and MG interactions alter redbanded stink bug oviposition with more egg masses oviposited on leaves in MG IV (79.4%) and more on pods in MG V (72.7%). Sex ratio increased to 1.4:1 females to males at R5, coinciding with peak oviposition. Only 29.9% of egg clusters in MG IV and 18.3% of egg clusters in MG V were oviposited in the upper 35 cm of the soybean canopy. Based on these results, sampling strategies and insecticide application placement for stink bugs may require modification.

## Figures and Tables

**Table 1:** Differences between soybean plant height and number of nodes during reproductive growth stages ^a^.

Growth Stage	Plant Height (cm) ± SE		No. of Nodes ± SE	
	MG IV	MG V	MG IV	MG V
R2	46.2 ± 0.7 ^d^	70.6 ± 0.8 ^e^	9.1 ± 0.1 ^d^	10.6 ± 0.1 ^d^
R3	66.0 ± 1.1 ^c^	91.7 ± 1.0 ^d^	11.4 ± 0.2 ^c^	12.0 ± 0.2 ^c^
R4	81.2 ± 1.6 ^b^	94.7 ± 0.9 ^cd^	13.7 ± 0.2 ^b^	12.4 ± 0.1 ^bc^
R5	86.6 ± 0.8 ^a^	97.3 ± 0.7 ^bc^	14.7 ± 0.1 ^a^	12.9 ± 0.1 ^b^
R6	89.2 ± 0.9 ^a^	99.7 ± 0.6 ^b^	14.8 ± 0.1 ^a^	12.7 ± 0.2 ^b^
R7	87.9 ± 1.1 ^a^	105.1 ± 1.0 ^a^	14.5 ± 0.1 ^a^	14.2 ± 0.2 ^a^

^a^ Means followed by same letter within columns are not significantly different (*p* > 0.05; Tukey’s Honestly Significant Difference (HSD)), MD = maturity group.

**Table 2:** Redbanded stink bug oviposition preference during reproductive growth stages ^a^.

Growth Stage	Mean % Egg Clusters ± SE	
	MG IV	MG V
R2	10.0 ± 9.0 ^bc^	1.9 ± 1.2 ^b^
R3	1.0 ± 0.6 ^c^	2.8 ± 1.3 ^b^
R4	6.0 ± 3.7 ^bc^	5.6 ± 3.1 ^ab^
R5	45.0 ± 12.0 ^a^	44.0 ± 14.4 ^a^
R6	23.7 ± 9.3 ^ab^	41.7 ± 16.9 ^ab^
R7	14.3 ± 9.0 ^b^	4.0 ± 2.0 ^ab^

^a^ Means followed by same letter within columns are not significantly different (*p* > 0.05; Tukey’s HSD).

## Abbreviations

The following abbreviations are used in this manuscript:

MG	Maturity Group
ANOVA	Analysis of Variance
HSD	Honest Significant Difference
